# Understanding compulsory community treatment across Australian jurisdictions: insights from three different data sources

**DOI:** 10.1192/bjo.2026.11019

**Published:** 2026-04-07

**Authors:** Claudia Bull, Anoushka Gaekwad, Jessica Madyson Layton, Bobbak Makooie, Alyshia Guan, Nithyashree Narayanan, Edwina Light, Steve Kisely

**Affiliations:** Queensland Centre for Mental Health Research, The Faculty of Health, Medicine and Behavioural Sciences, The University of Queensland, Australia; Princess Alexandra Hospital Southside Clinical Unit, Greater Brisbane Clinical School, Medical School, The Faculty of Health, Medicine and Behavioural Sciences, The University of Queensland, Australia; The ALIVE National Centre for Mental Health Research Translation, The University of Queensland, Australia; School of Public Health, Faculty of Medicine and Health, The University of Sydney, Australia; Metro South Addiction and Mental Health Services, Metro South Hospital and Health Service, Australia; Departments of Psychiatry, Community Health and Epidemiology, Dalhousie University, Canada

**Keywords:** Psychiatry and law, patients and service users, outpatient treatment, observational study, mental health services

## Abstract

**Background:**

Compulsory Community Treatment (CCT) is any intervention that mandates community psychiatric care. Despite Australia’s high use of CCT compared to other countries, there is no standardised national reporting framework, limiting transparency and comparability across jurisdictions.

**Aims:**

To determine rates of CCT orders per 100 000 population, individuals subject to CCT per 100 000 population and the proportion of all community mental healthcare contacts that were involuntary between 2016–2017 and 2023–2024. We also sought to identify and document differences in reporting practices across CCT reporting bodies.

**Method:**

Publicly available data were extracted from annual reports of state and territory Mental Health Review Tribunals or Civil and Administrative Tribunals, Offices of the Chief Psychiatrist and the Australian Institute of Health and Welfare. Rates of CCT orders per 100 000 population, individuals subject to CCT per 100 000 population and the proportion of all community mental healthcare contacts that were involuntary were calculated and compared across jurisdictions.

**Results:**

Marked differences were identified in CCT terminology, reporting scope and data completeness across jurisdictions and reporting bodies. Only three jurisdictions reported the number of individuals subject to CCT and none reported incidence data. Rates of CCT increased in most jurisdictions, except Western Australia, which showed a decline and the lowest rate of all jurisdictions. The proportion of involuntary community contacts ranged from 3 to 26% nationally.

**Conclusions:**

Australia’s fragmented CCT reporting landscape impedes accurate national monitoring. A standardised national CCT data-set that incorporates prevalence and incidence indicators is urgently needed to enable transparent, comparable reporting.

Compulsory Community Treatment (CCT) is a legal directive that mandates out-patient and community psychiatric care. Someone on CCT must have regular contact with mental health services, adhere to treatment plans and accept medication while living in the community.^1^ There is great debate around the use and effectiveness of CCT, particularly as CCT orders can remain in place for many years and the literature suggests CCT may violate human rights.^2^

## Variability in CCT effectiveness evidence

Central to the debate on CCT effectiveness is the variability in results arising from different study designs. Randomised controlled trials generally show that CCT does not reduce hospital readmissions, in-patient bed-days or symptoms over a 12-month period compared with voluntary community care.^3^ Contrastingly, observational and quasi-experimental studies report more favourable outcomes, including reduced mortality rates, but typically after a minimum duration of two years.^4^ Measures of CCT effectiveness may also differ depending on the specific outcomes assessed. Examples include service utilisation (e.g. hospital readmission rates and bed-days),^5^ clinical outcomes (e.g. psychiatric symptoms and functional status),^6^ medication adherence, criminal or aggressive behaviour,^7^ and mortality rates.^3^ However, due to marked differences in study designs, outcomes measured, populations under investigation and how biases (such as time-to-treatment bias) are addressed, the body of evidence remains ambiguous.

## Variability in CCT use nationally and internationally

Another area of concern is the considerable variability in CCT use. Though CCT is used in more than 75 jurisdictions globally, Australia and New Zealand report some of the highest rates of use.^8–10^ For example, Light^9^ showed that the rate of CCT orders made in 2016–2017 in Australia varied between 66.1 per 100 000 population in Queensland, to 108.4 per 100 000 population in Victoria and 112.5 per 100 000 population in South Australia. This far exceeds rates in comparable regions such as the Canadian provinces of Ontario and Saskatchewan and the state of New York in the US, where rates are approximately 2 per 100 000 population.^9,11^

In Australia, CCT is authorised under state and territory mental health legislation, with no overarching federal framework.^12^ While the specific statutory language varies, all jurisdictions embed CCT within their Mental Health Acts (MHAs) and confer decision-making authority to independent tribunals (e.g. Mental Health Review Tribunals (MHRTs) or Civil and Administrative Tribunals (CATs)), sometimes supplemented by short-term clinician-initiated orders.^13^ Across jurisdictions, the core criteria for CCT placement are broadly consistent, requiring the presence of a mental illness, a risk of serious harm or deterioration without treatment, impaired capacity to make treatment decisions, availability of effective treatment and satisfaction of a least-restrictive alternative test.^13^ Notwithstanding this apparent uniformity, jurisdictional nuance is evident. For example, in New South Wales (NSW), the MHA requires that for individuals with a prior diagnosis, there should be evidence of a history of refusal of, or non-adherence to, treatment before CCT placement can occur.^14^ Initiation pathways also differ, with applications variously permitted from authorised psychiatrists, treating clinicians, carers or guardians, though final orders are typically made or confirmed by a tribunal. Maximum durations of CCT orders vary by jurisdiction, ranging from short-term clinician-initiated orders (e.g. 42 days in South Australia) to tribunal-ordered placements of up to 12 months in most states, with provision for renewal.^14–16^

Australia’s high use of CCT has fuelled ongoing debates about the ethical ramifications of coercive treatment.^17^ Such practices, which inherently restrict personal liberty, necessitate stringent oversight to safeguard individual rights and prevent unjustified coercive therapy.^18^ The observed variability in CCT also raises significant ethical and practical concerns, suggesting that decisions regarding CCT may be shaped by structural and non-clinical determinants, such as resource distribution, institutional policies and the subjective risk thresholds of clinicians or tribunals, as opposed to the clinical needs of patients.^8^

## CCT reporting in Australia

In Australia, three main bodies report on CCT: (a) state and territory MHRTs or CATs, (b) state and territory Offices of the Chief Psychiatrist and (c) the Australian Institute of Health and Welfare (AIHW). No studies have systematically examined the availability or consistency of CCT data across these reporting bodies by jurisdiction, nor have they used these data to compare rates of CCT within, or across, Australian jurisdictions. Therefore, the aims of this study were two-fold. First, to determine rates of CCT orders per 100 000 population, rates of individuals subject to CCT per 100 000 population and the proportion of all community mental healthcare contacts that were involuntary using annual data reported by MHRTs/CATs, Offices of the Chief Psychiatrist and the AIHW across all Australian jurisdictions between 2016–2017 and 2023–2024. Second, to identify and document differences in reporting practices across the three reporting bodies. Together, this study provides the first comprehensive national synthesis of CCT across Australian jurisdictions.

## Method

### Study design

This study analysed secondary data in annual reports from MHRTs/CATs, Offices of the Chief Psychiatrist and the AIHW. The analysis covers all eight Australian jurisdictions (Queensland, South Australia, the Australian Capital Territory (ACT), Tasmania, Victoria, Western Australia, the Northern Territory and NSW) between 2016–2017 and 2023–2024. This time frame was selected to build on the work undertaken by Light.^9^ The authors assert that all procedures contributing to this work comply with the ethical standards of the relevant national and institutional committees on human experimentation and with the Helsinki Declaration of 1975, as revised in 2013. As this study utilised publicly available and de-identified data, ethics approval was not required. Participant consent was also not required.

### Data sources

Where available, we extracted raw data on the number of CCT orders made per year, the number of individuals subject to CCT and the proportion of all community mental healthcare contacts that were involuntary using data from annual MHRT/CAT, Offices of the Chief Psychiatrist and AIHW reports. Though South Australia, the Northern Territory, ACT and Tasmania do not have specific MHRT reports, some relevant mental health tribunal data were included in their annual CAT reports. These were included in the study to optimise data completeness and enhance cross-jurisdictional comparisons.

MHRTs (also referred to as Mental Health Tribunals) are independent statutory authorities, found in each state and territory, mandated to protect the rights of people who may require involuntary treatment for mental illness.^19–26^ They generally comprise a legal member (the Chairperson), a psychiatrist or other mental health practitioner and a community member with relevant lived experience. The role of the MHRT is to determine whether individuals should receive involuntary mental healthcare in the context of mental illness and/or intellectual disability. These decisions are made based on applications for CCT primarily made by clinicians from authorised mental health facilities.

Chief Psychiatrists are independent statutory officers found in each state and territory whose function is to protect the rights of individuals in authorised mental health facilities and make policies and practice guidelines for the staff working in these facilities.^27–31^ Though the scope of the Office of the Chief Psychiatrist differs slightly across jurisdictions, they are typically involved in matters related to involuntary mental health patients and the facilities they receive care in; the use of restrictive practices; and the processes, approvals and monitoring associated with treatment such as electroconvulsive therapy. Most Offices of the Chief Psychiatrist report publicly on these matters annually (except NSW and the Northern Territory), as well as on the administration of MHAs generally.

Lastly, the AIHW is an independent statutory government institution that operates at the national level.^12^ It collects and manages health and welfare data from state and territory health systems, including mental health data, and transforms it into accessible information to support improvements in policy and service delivery across Australia.^32^

### Data extraction and quality assurance: MHRT/CAT and Office of the Chief Psychiatrist data

Reports from MHRTs/CATs and the Offices of the Chief Psychiatrist were collected through online searches of the official websites for each jurisdiction, from which updated reports for each year were obtained. Where necessary, reporting bodies were contacted via email or phone to obtain data not available online. Following these measures, reports that remained unavailable were recorded as such.

Data from MHRT/CAT and Office of the Chief Psychiatrist reports were extracted from sections explicitly indicating that data related to CCT, although formatting varied considerably across reports and over time. From these sections, data on the number of CCT orders issued and/or the number of individuals subject to CCT were manually extracted (where available) into a structured Excel spreadsheet. Supplementary Material 1 (available at https://doi.org/10.1192/bjo.2026.11019) clarifies the source from which these data were extracted across each jurisdiction from 2016–2017 to 2023–2024. We also extracted data on the reporting body, jurisdiction, fiscal year of reporting, reasons for missing data and other important considerations (e.g. whether CCT was expressed as a point prevalence estimate or cumulative incidence rate). To ensure comparability across jurisdictions, terminology relating to CCT was extracted from each report. We did not extract data related to in-patient treatment orders, forensic treatment orders or combined order totals that could not be disaggregated. CCT orders that were not specifically related to mental health conditions, such as those for neurocognitive illness, including dementia, intellectual disability or acquired brain injury, were also excluded.

Where reports contained data breakdowns by CCT duration, only the total annual number of CCT orders made was extracted to maintain consistency across jurisdictions. For example, the South Australian Office of the Chief Psychiatrist reported CCT order numbers in the context of a two-tier system in which Level 1 orders were clinician-initiated over a short period (≤42 days) and Level 2 orders were tribunal-issued and extended over a longer period (≤12 months).^24,30^ We extracted the total number of CCT orders and the number of individuals subject to CCT irrespective of the ‘level’.

Following data extraction, compiled values were sent to the relevant MHRTs/CATs and the Offices of the Chief Psychiatrist for review as a quality assurance measure. This process served as a peer-review mechanism to enhance the accuracy and consistency of the final data-set. We also compared our 2016–2017 data extract to the data reported by Light,^9^ confirming its validity.

### Data extraction and quality assurance: AIHW data

Data from the AIHW were directly extracted from the ‘Involuntary Treatment in Mental Health Care’ section of the AIHW’s *Mental Health Care Services in Australia* online reporting portal (specifically Figure Invol.1: Involuntary treatment in Australia).^12^ This figure presents the annual number and proportion of all community mental healthcare contacts that were involuntary, stratified by year and jurisdiction. Data were extracted from the interactive online tables across the years 2016–2017 to 2022–2023 into a structured Excel spreadsheet. Data for 2023–2024 were not reported at the time of analysis.

### Data analysis

#### Cross-jurisdictional heat map

To compare reporting practices across jurisdictions, a heat map was constructed using data from the 2023–2024 MHRT/CAT and the Office of the Chief Psychiatrist reports. Eleven criteria were assessed based on whether the following were reported: (a) community and in-patient orders reported separately; (b) total number of CCT orders made (i.e. order events); (c) total number of involuntary orders made (community and in-patient); (d) number of individuals on existing CCT orders (prevalence); (e) number of individuals on new CCT orders (incidence); (f) number of MHRT/CAT matters; (g) number of MHRT/CAT sittings and/or hearings; (h) number of MHRT/CAT reviews; (i) number of MHRT/CAT inquiries; (j) number of MHRT/CAT appeals; and (k) whether reports were publicly accessible. Notably, this list does not represent every variable within the annual reports. It was developed to capture the core elements most relevant to documenting CCT for this study.

Each of the eleven reporting criteria were then coded using a traffic light system to reflect the clarity and completeness of reporting. Green indicated that the criterion was explicitly reported and clearly defined; yellow indicated that the criterion was only partially reported across years and/or defined ambiguously; and red indicated the criterion was not reported. Coding decisions were based on direct review of the MHRT/CAT and Office of the Chief Psychiatrist reports and were reviewed by multiple authors to ensure accuracy.

#### Calculations of rates per 100 000 population

Using the raw data extracted from MHRT/CAT and Office of the Chief Psychiatrist reports, rates of CCT orders per 100 000 population and individuals subject to CCT per 100 000 population were calculated. Population data for each state and territory for the relevant year were obtained from the Australian Bureau of Statistics (ABS) to enable per 100 000 population calculations.^33^ The following formulae were used, emulating the method used by Light:^9^


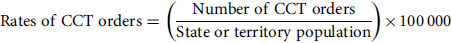




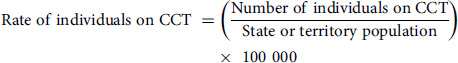




Rates of CCT orders per 100 000 population and individuals subject to CCT per 100 000 population were tabulated and graphically presented using bar graphs. The proportion of all community mental healthcare contacts that were involuntary (AIHW data) were also tabulated and graphically presented. All results and standard error bars were plotted in Microsoft Excel (Version 2602 for Windows, Microsoft Corporation, Santa Rosa, California, USA; https://www.microsoft.com/en-us/microsoft-365/download-office).

## Results

### CCT terminology

There was considerable variation in the terminology used for CCT across Australian jurisdictions. Table 1 shows that Western Australia, NSW and South Australia use the term Community Treatment Order (CTO); Northern Territory uses the term Community Management Orders (CMO); Tasmania and Victoria use Treatment Orders; Queensland uses Treatment Authorities; and the ACT uses Psychiatric Treatment Orders (PTO). PTOs are the only order that does not have a defined setting according to the ACT MHA of 2015;^34^ it may be based in the community or in-patient setting. There have also been changes in terminology over time. For instance, prior to 2017, Treatment Authorities in Queensland were called Involuntary Treatment Orders. The AIHW reports on the proportion of all community mental healthcare contacts that were involuntary (i.e. contacts by individuals on CCT).

**Table 1:** Current terminology for Compulsory Community Treatments across Australian jurisdictions and nationally

Jurisdiction	Terminology
Queensland	Treatment Authority
NSW	Community Treatment Order
Victoria	Treatment Order
South Australia	Community Treatment Order
Tasmania	Treatment Order
Western Australia	Community Treatment Order
Northern Territory	Community Management Order
ACT	Psychiatric Treatment Order
National (AIHW)	Proportion of all community mental healthcare contacts that were involuntary

NSW, New South Wales; ACT, Australian Capital Territory; AIHW, Australian Institute of Health and Welfare.

### Cross-jurisdictional heat map

**Fig. 1:**
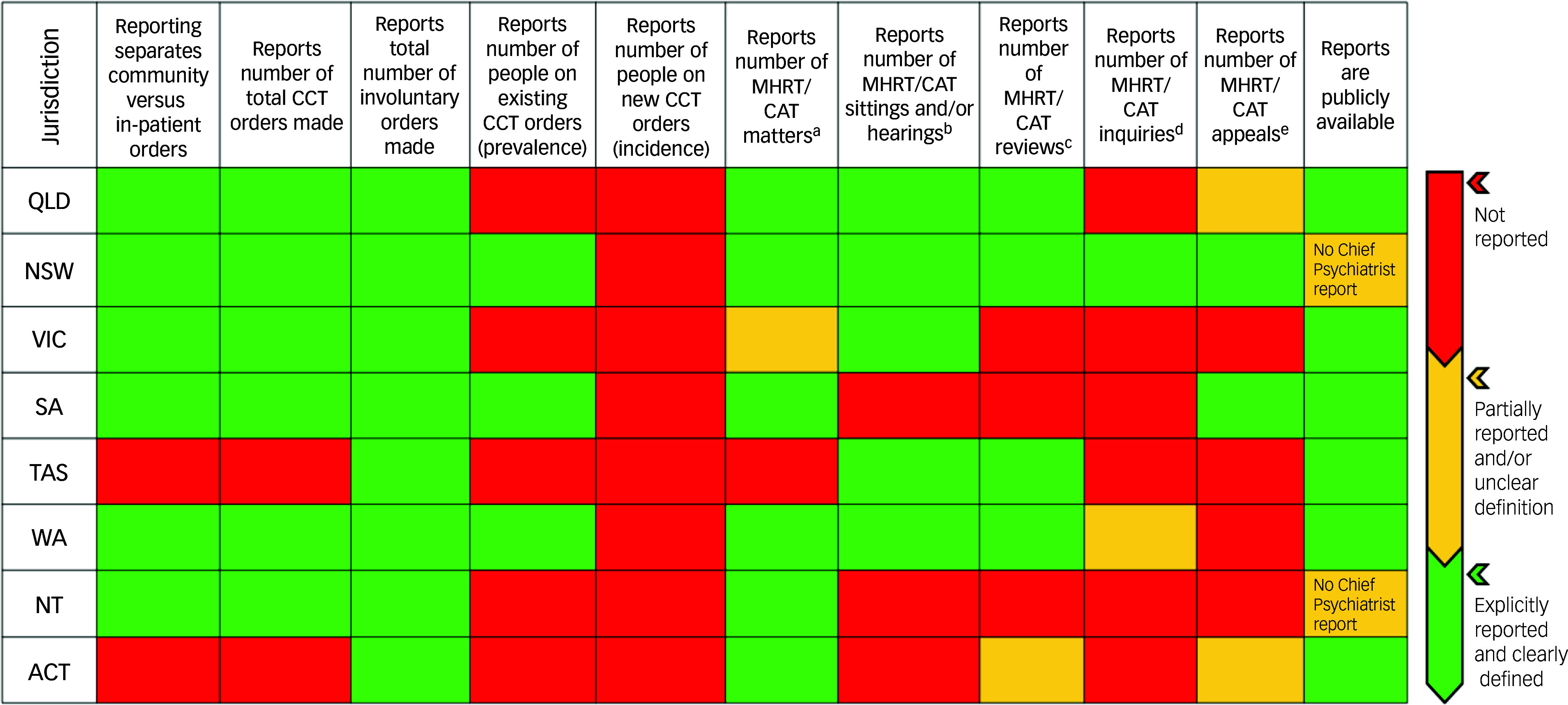
Cross-jurisdictional heat map comparing reporting practices in 2023–2024 MHRT/CAT and the Office of the Chief Psychiatrist report. a. Matters refer to any issue or case that comes before the MHRT/CAT for decision; b. Sittings are scheduled sessions or blocks of time during which the MHRT/CAT conducts hearings, which in turn, are formal proceedings where the MHRT/CAT considers evidence and submissions before deciding on a matter; c. Reviews are statutory reconsiderations of a person’s community, inpatient or forensic mental health order status which are required at specific intervals by MHA law; d. Inquiries are a specific form of review triggered when certain conditions are met that might jeopardise patient rights; e. Appeals represent a challenge to a decision that has already been made and is initiated by the involuntary patient or their representative. CCT, Compulsory Community Treatment; MHRT/CAT, Mental Health Review Tribunal/Civil or Administrative Tribunals; QLD, Queensland; NSW, New South Wales; VIC, Victoria; SA, South Australia; TAS, Tasmania; WA, Western Australia; NT, Northern Territory; ACT, Australian Capital Territory.

Figure 1 shows the variation in scope and content of CCT reporting across Australian jurisdictions in the context of reports from MHRTs/CATs and the Offices of the Chief Psychiatrist. Each jurisdiction reported the total number of involuntary orders made (combining community and in-patient categories), though Tasmania and the ACT did not report community and in-patient orders separately. Only NSW, South Australia and Western Australia reported the number of people on CCT and no jurisdictions reported the number of new (incident) people on CCT. Most jurisdictions reported on the number of CCT-related matters at MHRTs or CATs, but only NSW reported on the number of hearings/sittings, reviews, inquiries and appeals. Neither NSW nor the Northern Territory have publicly available Office of the Chief Psychiatrist reports.

#### Rates of CCT orders per 100 000 population using data from MHRT/CAT and Office of the Chief Psychiatrist reports

**Fig. 2:**
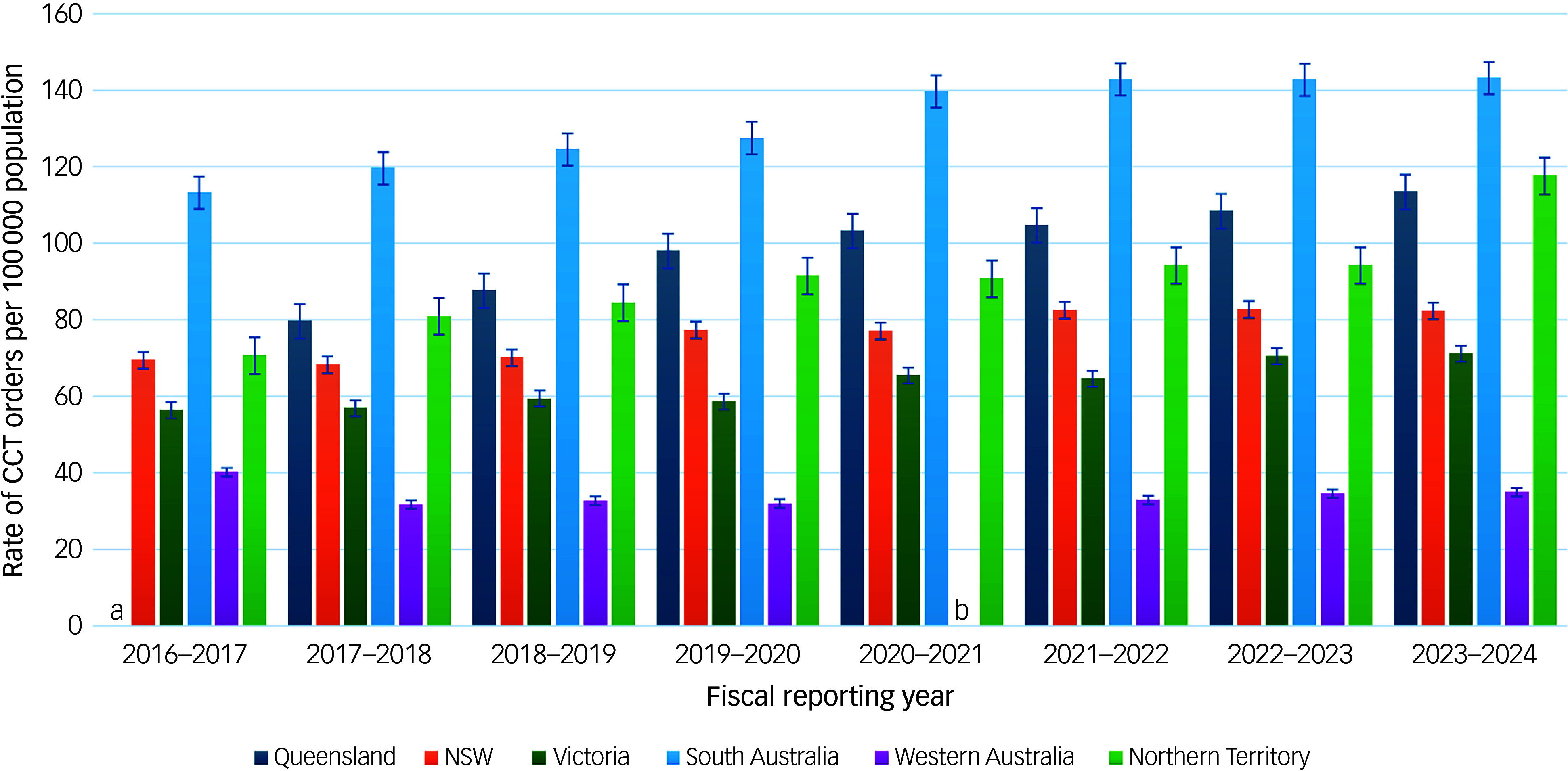
Rates of CCT orders per 100 000 population. a. CCT orders not reported separately from other types of orders in 2016–2017 only; b. CCT orders not reported separately from other types of orders in 2020–2021 only; Tasmania and ACT omitted as they do not report CCT order data separately from other types of orders. CCT, Compulsory Community Treatment; NSW, New South Wales; ACT, Australian Capital Territory.

Figure 2 and Table 2 show that all jurisdictions, with the exception of Western Australia, demonstrated increased rates of CCT orders per 100 000 population from 2016–2017 to 2023–2024. Western Australia recorded the lowest rates and was the only jurisdiction to show an overall absolute decline between 2016–2017 and 2023–2024. In contrast, Queensland, NSW, Victoria, South Australia and the Northern Territory all demonstrated consistent upward trends in rates across the same period.

**Table 2:** Rates of Compulsory Community Treatment orders per 100 000 population

Jurisdiction	2016–2017	2017–2018	2018–2019	2019–2020	2020–2021	2021–2022	2022–2023	2023–2024
Queensland	–^a^	79.6	87.6	98.0	103.2	104.7	108.4	113.4
NSW	69.4	68.2	70.1	77.3	77.1	82.5	82.7	82.3
Victoria	56.4	56.9	59.4	58.6	65.4	64.6	70.5	71.1
South Australia	113.2	119.6	124.5	127.5	139.7	142.8	142.7	143.2
Tasmania^b^	–	–	–	–	–	–	–	–
Western Australia	40.2	31.7	32.7	32.0	–^c^	32.9	34.6	34.9
Northern Territory	70.6	80.9	84.5	91.5	90.7	94.2	94.2	117.6
ACT^b^	–	–	–	–	–	–	–	–

NSW, New South Wales; ACT, Australian Capital Territory.

a. CCT orders not reported separately from other types of orders in 2016–2017 only.

b. CCT orders not reported separately from other types of orders.

c. CCT orders not reported separately from other types of orders in 2020–2021 only.

#### Rates of individuals subject to CCT per 100 000 population using data from MHRT/CAT and Office of the Chief Psychiatrist reports

**Fig. 3:**
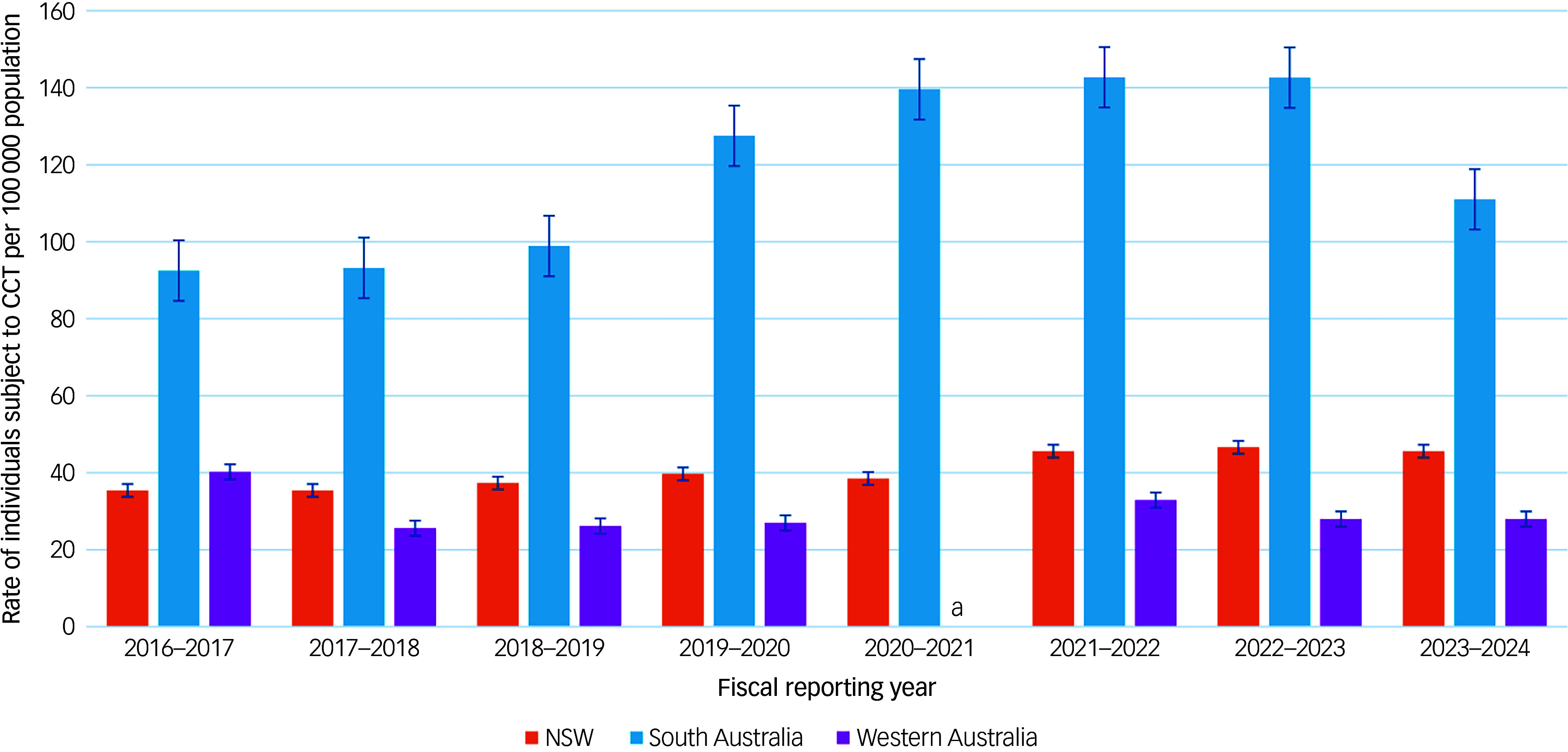
Rates of individuals subject to CCT per 100 000 population. a. Number of individuals subject to CCT not reported separately from other types of orders in 2020–2021 only; Queensland, Victoria, Tasmania, Northern Territory and ACT omitted as they do not report data on the number of individuals subject to CCT. CCT, Compulsory Community Treatment; NSW, New South Wales; ACT, Australian Capital Territory.

Figure 3 and Table 3 show trends in the rates of individuals subject to CCT per 100 000 population. These trends are broadly consistent with the rates of CCT orders per 100 000 population, though notably, only three jurisdictions reported data on the number of individuals subject to CCT. NSW and South Australia demonstrated overall increasing trends, with South Australia reporting the highest rates among all three jurisdictions. Comparably, Western Australia demonstrated an overall decrease over time and the lowest rates among all three jurisdictions.

**Table 3:** Rates of individuals subject to Compulsory Community Treatment (CCT) per 100 000 population

Jurisdiction	2016–2017	2017–2018	2018–2019	2019–2020	2020–2021	2021–2022	2022–2023	2023–2024
Queensland^a^	–	–	–	–	–	–	–	–
NSW	35.4	35.4	37.3	39.7	38.5	45.6	46.6	45.6
Victoria^a^	–	–	–	–	–	–	–	–
South Australia	92.5	93.2	98.9	127.5	139.6	142.7	142.6	111.0
Tasmania^a^	–	–	–	–	–	–	–	–
Western Australia	40.2	25.6	26.2	27.0	–^b^	32.9	28.0	28.0
Northern Territory^a^	–	–	–	–	–	–	–	–
ACT^a^	–	–	–	–	–	–	–	–

NSW, New South Wales; ACT, Australian Capital Territory.

a. No data reported on the number of people subject to CCT.

b. Number of individuals subject to CCT not reported separately from other types of orders in 2020–2021 only.

#### Trends in the proportion of all community mental healthcare contacts that were involuntary using data from AIHW reports

**Fig. 4:**
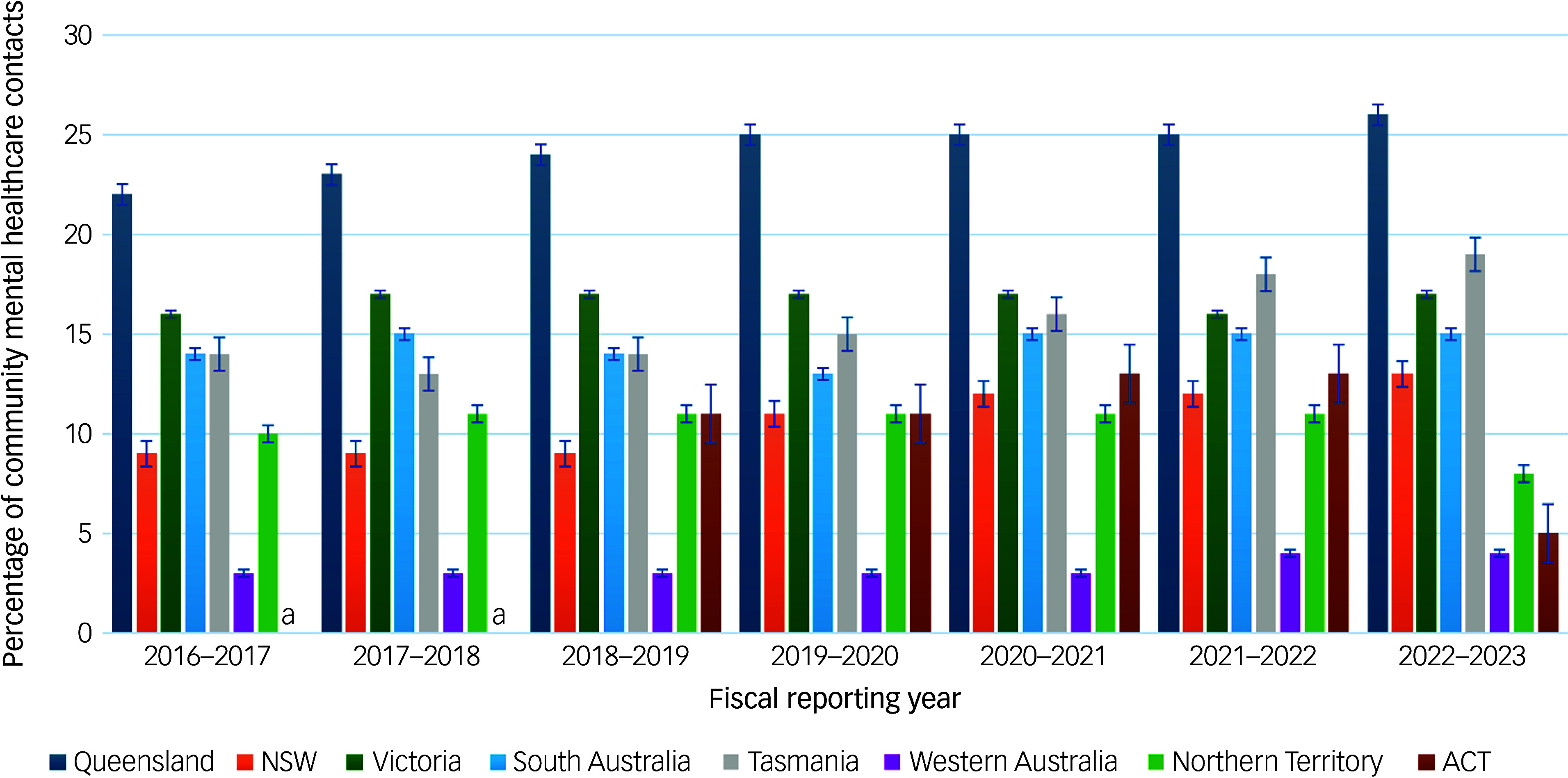
Proportion of all community mental healthcare contacts that were involuntary, 2016–2017 to 2022–2023. a. Data for 2016–2017 and 2017–2018 were excluded for ACT due to a change in the reporting system, which made these years’ data not directly comparable. NSW, New South Wales; ACT, Australian Capital Territory.

Figure 4 and Table 4 illustrate the proportion of all community mental healthcare contacts that were involuntary (i.e. contacts by individuals on CCT) for each jurisdiction between 2016–2017 and 2022–2023. The proportion of involuntary contacts showed a steady or upward trend over time in most jurisdictions (except the Northern Territory and ACT), with Tasmania increasing the most by 5%, followed by a 4% increase in both NSW and Queensland. The proportion of all community mental healthcare contacts that were involuntary varied widely across jurisdictions, with the lowest consistently in Western Australia (3–4%) and the highest in Queensland (peaking at 26% in 2022–2023). The proportions in other jurisdictions generally ranged between 9 and 25%. Between 2016–2017 and 2021–2023, the proportion of involuntary community contacts ranged from 3 to 26% across all jurisdictions.

**Table 4:** Proportion of all community mental healthcare contacts that were involuntary, 2016–2017 to 2022–2023

Jurisdiction	2016–2017, %	2017–2018, %	2018–2019, %	2019–2020, %	2020–2021, %	2021–2022, %	2022–2023, %
Queensland	22	23	24	25	25	25	26
NSW	9	9	9	11	12	12	13
Victoria	16	17	17	17	17	16	17
South Australia	14	15	14	13	15	15	15
Tasmania	14	13	14	15	16	18	19
Western Australia	3	3	3	3	3	4	4
Northern Territory	10	11	11	11	11	11	8
ACT	–^a^	–^a^	11	11	13	13	5

NSW, New South Wales; ACT, Australian Capital Territory.

a. Data for 2016–2017 and 2017–2018 were excluded for ACT due to a change in the reporting system, which made these years’ data not directly comparable.

## Discussion

Transparent and consistent reporting should be an essential policy requirement to document the use of CCT, assess the effectiveness of its application and ensure adequate safeguards are in place to maintain human rights. This study represents the first national comparison of CCT data across all Australian jurisdictions, integrating information from three reporting bodies: MHRTs/CATs, Offices of the Chief Psychiatrist and the AIHW. By analysing data from 2016–2017 to 2023–2024, this study identified several inconsistencies in the reporting of CCT, not only between jurisdictions but also between reporting bodies within the same jurisdiction, highlighting the fragmented nature of Australia’s current reporting systems.

A key finding of this study is the complexity of constructing a comparable metric of CCT use across the three reporting bodies. Sources captured overlapping dimensions of CCT, using different denominators, definitions and reporting structures. The coexistence of these disparate reporting practices means that researchers and policymakers must reconstruct national trends from non-equivalent data-sets that vary in scope, format and terminology, to approximate the scale of CCT. The absence of a consistent national definition of CCT further complicates the matter, as this suggests jurisdictions use different legislative thresholds and renewal mechanisms. This fragmentation limits the accuracy of cross-jurisdictional comparisons and hampers efforts to evaluate the effectiveness of CCT, which has significant ethical implications for policymaking and healthcare delivery.

While there were several differences across reporting sources, there were also some similarities. For instance, there were some jurisdictions such as Queensland that showed higher rates than most other jurisdictions irrespective of reporting source. By contrast, Western Australia demonstrated both the lowest CCT rates per 100 000 population and the lowest proportions of community mental healthcare contacts that were involuntary using AIHW data; an internally consistent pattern that may suggest comparatively limited use of CCT across the state. It is therefore possible that while differences in definition and scope preclude direct comparison, the directional trends suggest that the underlying drivers of CCT, such as service culture, legislation and system capacity, are still evident in the different data sources. Indeed, service culture, legislation and system capacity may also influence whether CCT is operationalised primarily as a least-restrictive alternative to in-patient compulsory care (consistent with MHA intent) or as a more preventive mechanism aimed at managing perceived risk and reducing deterioration in the community.^17,18^ In this sense, variation in CCT use cannot be understood in isolation, as different jurisdictional thresholds for in-patient involuntary admission, bed availability and community service capacity all shape the balance between community and in-patient involuntary treatment.^11,12,35^

The differences in CCT use across Australian jurisdictions in the current study mirror findings from our previous research that used bespoke AIHW data on the proportions of people in contact with community mental health services who were receiving involuntary care.^8^ This is in contrast to the publicly available AIHW data used in this study that reported on the proportion of care episodes, not people, that involved involuntary care. In that study, South Australia had the highest rates of CCT use (17.6%) followed by Queensland (16.0%), Victoria (13.8%) and NSW (8.0%), respectively.^8^

The findings of the current study have significant national and international implications for the standardised reporting of CCT. While the AIHW’s national reporting on the proportion of community mental healthcare contacts that are involuntary provides a broad indicator of coercive practices, it does not capture the number of individuals subject to CCT, nor the frequency with which these orders are made.^12^ Consequently, the data reflect service activity rather than the scope or intensity of CCT and fails to convey its individual-level impact. In contrast, MHRTs/CATs and the Offices of the Chief Psychiatrist hold both the legal authority and administrative infrastructure necessary to systematically monitor CCT activity, review decisions and uphold accountability for individual rights. To ensure transparency and comparability, future reporting should prioritise the development of a standardised national CCT data-set. This would allow data to be meaningfully compared across jurisdictions and over time, capturing both prevalence (i.e. the number of individuals on existing CCT orders) and incidence (i.e. the number of individuals on new CCT orders). Such a data-set should also uniformly capture information on sociodemographic and clinical characteristics (e.g. age, sex, diagnosis, First Nations status, cultural and linguistic diversity) known to be associated with CCT placement in Australia.^5,8,36^ Given the various bodies involved in the collection and reporting of these data, federal intervention would be required, as would strong collaboration between the AIHW and state/territory-based Offices of the Chief Psychiatrist and MHRTs/CATs. Similar to the development of the Child Protection National Minimum Data Set in response to the *National Framework for Protecting Australia’s Children 2009–2020*,^37^ a National Framework for involuntary mental healthcare – or more specifically for CCT – would be required to support the development of a nationally standardised CCT data-set and ensure jurisdictional action and accountability. Establishing this level of standardisation is essential not only to reduce duplication of effort (such as the extensive cross-source reconciliation required in the present study) but also to strengthen oversight of what is ultimately an issue of human rights, safety and quality in mental healthcare.

The need for standardised reporting across jurisdictions and countries has been highlighted by work on coercive practices in in-patient psychiatric care.^35^ This covered publicly available data on involuntary hospital admission, seclusion, mechanical and physical restraint, as well as involuntary medication from Australia, England, Germany, Ireland, Japan, The Netherlands, New Zealand, the USA and Wales. As in this study, measures were highly variable and poorly reported, highlighting the need for standardised reporting to enable meaningful comparisons over time and place.

In addition, research is needed to understand whether legislative requirements partly explain the variation in CCT use across Australia. Each jurisdiction’s MHA defines different thresholds, durations and renewal procedures for CCT, which likely contributes to the observed discrepancies in rates.^38^ Thus, a comparative legislative review of Australian MHAs may identify which legal provisions influence the frequency and duration of CCT, their reporting and whether some jurisdictions strike a greater balance between upholding human rights while providing involuntary therapeutic intervention.

### Strengths and limitations

This study offers several key strengths that enhance its contribution and credibility to the existing literature. It is the first national comparison of CCT data across jurisdictions, integrating information from three reporting bodies. This enabled a triangulated and comprehensive view of CCT use which no single data-set could provide, revealing differences in scope, terminology and reporting focus that have not previously been examined. Moreover, by analysing data from 2016–2017 to 2023–2024, the study captures evolving practice and illuminates important temporal trends and regional variations in CCT use. It also builds on and extends over 20 years’ worth of research trying to understand rates and patterns of CCT use.^1,9,39^

Another strength of the current study is that data extraction underwent multiple layers of quality assurance, including cross-referencing with jurisdictional reporting bodies and peer review within the research team. This sought to optimise the accuracy, consistency and validity of the study’s results. Together, these strengths support the robustness and interpretability of the findings.

There are, however, important limitations that need to be considered. Notably, the limitations of this study mirror the inadequacies of the CCT reporting landscape. First, direct comparisons between jurisdictions were limited to only a subset of outcomes (e.g. rates of CCT orders per 100 000 population) because of discrepancies in reporting practices across the different reporting bodies and jurisdictions. These differences likely reflect contrasting legal and administrative perspectives, as well as variations in the type of estimates reported (e.g. point prevalence versus cumulative incidence). Moreover, important sociodemographic and clinical characteristics of the populations placed on CCT (e.g. age, sex, mental illness diagnosis, First Nations status, cultural and linguistic diversity) largely go unremarked upon in all reports (with the exception of South Australia), despite the known associations these features have with CCT placement in Australia.^5,8,36^ Therefore, our results should be interpreted cautiously. Second, while the Office of the Chief Psychiatrist reports typically documented the number of existing CCT orders, MHRT/CAT reports captured tribunal-related activities such as hearings. As a single person may appear before a tribunal multiple times in a reporting year, these figures do not equate to unique individuals. Consequently, each reporting body may report a different ‘rate’ of CCT for the same jurisdiction, making cross-source comparisons complex.

Third, year-to-year changes should be interpreted cautiously in smaller jurisdictions such as the Northern Territory, where modest absolute differences in CCT order numbers can produce substantial changes in rates per 100 000 population. This may reflect statistical instability rather than substantive shifts in practice. Interpretation of South Australia’s rates should also be approached with caution, as its Level 1 and 2 CCT order reporting system may have resulted in double-counting and consequently inflated reported figures compared with other jurisdictions. However, this is unlikely to be the only explanation for South Australia’s high rates of CCT, given that they also had the highest rates of three other states (Queensland, Victoria and NSW) in our previous research, irrespective of whether the order was Level 1 or 2.^8^

Fourth, in combining CCT counts from MHRT/CAT and Office of the Chief Psychiatrist reports to generate a standard measure by jurisdiction and year, some distinctions between the two data sources may have been lost. Thus, the combined measure should be regarded as a general indicator of CCT use rather than a precise estimate. Finally, the AIHW has noted several data completeness and comparability issues that may have influenced results. These include a change in information systems for NSW between 2016–2017 and 2018–2019 and periods of industrial action in Tasmania during 2018–2019, parts of 2019–2020 and 2021–2022 and in Victoria during 2016–2017, 2020–2021 and 2021–2022.^12^ ACT data were also excluded prior to 2018 due to changes in data collection processes.^12^ Additionally, the AIHW does not distinguish between individuals who were subject to community and forensic orders when reporting on the proportion of all community mental healthcare contacts that were involuntary. However, forensic orders represent a small number of CCT orders and are unlikely to have had a significant impact on these data.

In summary, CCT remains a contentious issue due to ambiguous evidence of effectiveness and concerns about infringement of fundamental human rights. This study is the first to compare CCT use across Australian jurisdictions using publicly available data from MHRTs/CATs, Offices of the Chief Psychiatrist and the AIHW. Findings demonstrated significant variation in terminology, rates of CCT orders and documentation of CCT use across jurisdictions and data sources. These results unequivocally demonstrate the critical need for a standardised national CCT data-set that allows data to be meaningfully compared across jurisdictions and over time. This will be crucial to ensuring appropriately targeted psychiatric care can be provided to those who need it. Given the absence of standardised measures in international comparisons of CCT use, it is likely that our findings have implications beyond Australia.

## Supporting information

Bull et al. supplementary material Bull et al. supplementary material

## Data Availability

Data availability is not applicable to this article as no new data were created or analysed in this study.

## Supplementary material

The supplementary material is available online at https://doi.org/10.1192/bjo.2026.11019
